# Current state and the support system of athlete wellbeing in Japan: The perspectives of the university student-athletes

**DOI:** 10.3389/fpsyg.2022.821893

**Published:** 2022-10-13

**Authors:** Yoriko Noguchi, Chisato Kuribayashi, Taisuke Kinugasa

**Affiliations:** ^1^Japan Institute of Sports Sciences, Japan High Performance Sports Center, Japan Sport Council, Tokyo, Japan; ^2^Faculty of Physical Education, Tokyo Women’s College of Physical Education, Tokyo, Japan

**Keywords:** psychological/mental wellbeing, mental health, athlete health, high-performance sports, COVID-19, sport policy, athlete support system, Asia

## Abstract

The optimization of athletes’ wellbeing has been increasingly considered essential both in the academic and practical fields of high-performance sports. Various organizations, such as the International Olympic Committee, have highlighted its importance, particularly mental health. Moreover, the increased attention to athlete wellbeing in sport policy debates at the national level has led to the development and implementation of a support system for athletes’ mental wellbeing in some countries. Nevertheless, the literature is limited to understanding the case of Japan. Interestingly, only 0.8% of the literature is available on “athlete” and “wellbeing” in Japanese compared to English journals up to 2019. Therefore, the purpose of this study was to identify (a) the current state of wellbeing of Japanese university student-athletes, (b) the level of knowledge about athlete wellbeing, and (c) the athletes’ perception of the availability of wellbeing support in the national sports federations, (d) the athlete experience of support services, and develop the types of national support athletes expect and need from the government and national sports federations in the future. As a pilot study, a total of 100 Japanese university student-athletes (43 male, 57 female) from 17 Olympic and seven Paralympic sports completed an online survey. Consequently, the state of their wellbeing was self-perceived as good in all dimensions (i.e., physical, mental, educational, organizational, social, and financial). Moreover, the results showed low recognition of the term “athlete wellbeing” and a lack of knowledge of the availability and accessibility of appropriate support services. The results also showed that Japanese university student-athletes rarely seek help from experts, while 45% indicated “no one” to talk to. Interestingly, however, most athletes considered each dimension of wellbeing important in relation to their performance development. Based on the results, it is necessary to develop an education program, guidelines, and detection systems and improve information accessibility. Given that this pilot study’s validity, reliability, and feasibility were verified, further studies should focus more on the wellbeing of Japanese elite athletes in high-performance sports (i.e., Olympic and Paralympic athletes).

## Introduction

The optimal and holistic development as a human being is considered important for athletes to achieve their maximum potential both in performance and life after their athletic careers (Wylleman, 2019). Although participation in sports and physical activity benefits one’s health and mental wellbeing in many ways (Biddle et al., 2015), pursuing excellence in high-performance sports is associated with various factors that may pose threats to the holistic wellbeing of athletes (MacAuley, 2012; Gouttebarge et al., 2019; Giles et al., 2020).

Given those risks in a highly competitive environment, optimizing athlete wellbeing, particularly mental health, has received considerable attention in high-performance sports academic, political, and practical fields. The increase in interest might be triggered by some high-profile athletes openly and publicly discussing their challenges with mental health and wellbeing (Heaney, 2021). In the period between 2018 and 2020, several sporting organizations published consensus statements on athletes’ mental health, including the International Olympic Committee (IOC) (Moesch et al., 2018; Schinke et al., 2018; Gorczynski et al., 2019; Reardon et al., 2019; Van Slingerland et al., 2019; Henriksen et al., 2020).

At the same time, several national governments and sports organizations have conducted investigations and developed policies to guide the nation to promote and support athletes’ mental wellbeing at a system level (Canadian Olympic Committee, 2015; Department for Digital, Culture, Media and Sport, 2018; English Institute of Sport, 2019; Australian Institute of Sport, 2020; High Performance Sport New Zealand, 2021). To operationalize the policy into practice, some leading countries have launched teams responsible for establishing and implementing the national support system and programs, mostly at the high-performance sports centers, as an integral part of athlete development. Those support frameworks appeared to include some of the common approaches proposed by Purcell et al. (2019): (a) providing support for athletes to equip them with a range of skills to self-manage distress, (b) educating key stakeholders (e.g., coaches, science and medicine practitioners, support service providers, etc.) in a high-performance environment to better understand and respond to symptoms regarding mental health and wellbeing, and (c) establishing multi-disciplinary teams and/or professionals to better support and manage prevention and reaction to athletes’ problems with mental health and wellbeing.

Despite mounting literature and practical implementation of policies to support athlete wellbeing, there are several limitations associated with athlete wellbeing. First, the majority of research have focused on athletes’ physical and psychological/mental wellbeing, even in the last 2 years (Biggins et al., 2020; Schary and Lundqvist, 2021; Jovanovic et al., 2022). Thus, there is little to know about the athlete’s wellbeing from a holistic perspective. Furthermore, Giles et al. (2020) argued that evidence-based intervention in athlete wellbeing is limited due to methodological and conceptual issues. Lundqvist (2011) also claimed that “wellbeing is treated as an unspecific variable, inconsistently defined and assessed using a variety of theoretically questionable indicators (p.118).” These methodological and conceptual issues associated with athlete wellbeing, therefore, make it difficult to carry out evidence-based interventions in practice (Giles et al., 2020). Moreover, despite most studies having been conducted in Western countries, there is still little information available about other regions, including Asia (Reardon et al., 2019). Additional research, therefore, contributes to knowledge in this area, particularly in developing the support policy and framework that could be operationalized in practice.

Japan earned 27 gold medals and 58 total medals in the Tokyo 2020 Olympic Games, placing them in the top three nations for gold medals, which were the best results ever. Since the development of sports has become the responsibility of the government due to the enactment of the Basic Act on Sport in 2011 (Ministry of Education, Culture, Sports, Science and Technology, 2011), the landscape of Japanese high-performance sports has dramatically changed at all levels, such as policies, systems, the structure, and programs. However, there had been little discussion about athlete mental health and/or wellbeing until the COVID-19 pandemic struck, resulting in the Tokyo 2020 Games being postponed by 1 year. In fact, Kinugasa et al. (2021) reported that only 14 articles were available on “athlete” and “wellbeing” in the Japanese language; it was only 0.8% of those in English journals up to 2019. However, gradually more focus is being directed toward athletes’ mental health, that is, a state of mental wellbeing. For example, Tsuchiya et al. (2021) argued the need for support for athletes’ mental health by reporting the positive correlation with a psychological stress response to COVID-19.

To contribute to Evidence-Based Policy Making (EBPM) in the high-performance sports field, the Japan Sport Council (JSC) launched a new research group in social sciences at the Japan Institute of Sports Sciences (JISS), a part of the Japan High Performance Sport Center (HPSC) (Kukidome and Noguchi, 2020). Given the limited evidence available in the field of athlete wellbeing in Japan, the group initiated the research project to provide some evidence to support the policy development into operationalization in Japan–that is, a pilot study with university student-athletes aiming to reveal (a) the current state of wellbeing of Japanese university student-athletes, (b) the level of knowledge about athlete wellbeing, (c) the student-athletes’ perception of the availability of wellbeing support in the national sports federations, and (d) the student-athletes experience of support services on wellbeing, and develop the types of national support student-athletes expect and need from the government and national sports federations in the future.

## Materials and methods

### Participants

The participants for the pilot study included 100 Japanese university student-athletes (43 male, 57 female) aged from 20 to 25 years (*M* = 21.3, SD = 1.2). The sample was limited to student-athletes who attend either undergraduate or postgraduate programs and belonged to the university’s Athletic Department, participating in sports in an official event of the Tokyo Olympic and Paralympic Games 2020. The participants represented 18 Olympics (baseball and softball, basketball, athletics, volleyball, football, badminton, tennis, swimming, table tennis, archery, handball, judo, rhythmic gymnastics, rugby sevens, artistic gymnastics, karate, surfing, and water polo), and seven Paralympic sports (para-table tennis, para-badminton, para-swimming, para-archery, boccia, para-athletics, and para-judo). The participants were grouped into two categories: “elite” for those who have competed in international competitions representing Japan, including five serial medalists (36.0%), and “sub-elite” for the rest (64.0%). 11% of the participants were carded athletes in national (*n* = 1), senior (*n* = 4), youth (*n = 3*), and junior (*n = 1*) categories for less than 1 year (33.3%), 1–3 years (44.4%), and 4–6 years (22.2%).

### Measures

Given that this pilot study was specifically designed for the initial investigation to capture the general trends of student-athlete wellbeing in Japan with the aim of providing evidence for developing the support system within the country, the instrument was self-developed in the Japanese language. To maintain the holistic nature of wellbeing, we developed the instrument in accordance with the Holistic Athlete Career Model (Wylleman, 2019). To validate this 48-item instrument, we used the Delphi method (Hsu and Sanford, 2007) by eight psychologists and social scientists with an excellent understanding of athlete wellbeing. The instrument was resurveyed until the experts reached a consensus (100% agreement by the eight experts), and the content validity and feasibility of the instrument were verified through this process. The reliability of the instrument was tested by administering the same instrument twice to the same 38 respondents, the participants within 1 week, and calculating the intraclass-correlation coefficient (ICC). Test-retest reliability of the instrument was found to be good (*r* = 0.7 ± 0.3) (Hopkins, 2000).

#### Demographic information

The measurement consisted of 11 items to gather demographic information about the participants. These items included gender, age, place of living, working/educational status, sport type, the number of years played in their main sport, organizational type, carded category, the number of years played in their carded category, and the best performance record in their sport.

#### Awareness of and state of athletes’ wellbeing

As “athlete wellbeing” is a relatively new concept in Japan, one item was included to understand the level of awareness in student-athletes. In addition, it comprised seven Likert-scale items to measure the state of wellbeing in each dimension (i.e., physical health, psychological health, balance with education/and or work, interpersonal relationships, organizational environment, financial security and stability, and legal security and safety). Their states of wellbeing in each dimension were asked over the past 3 years to account for COVID-19 spread mostly in 2020 in Japan, and a 5-point scale was used in most of the items (e.g., 1 = *very good*, 2 = *somewhat good*, 3 = *not so good*, 4 = *not good at all*, 5 = *not sure*). Furthermore, in order to take the degree of influence of COVID-19 into consideration, another seven items were added (e.g., *Does COVID-19 have more influence on your wellbeing than usual before the pandemic?*).

#### Influence and importance of wellbeing in relation to athlete performance

A total of 12 items were included in the instrument to reveal the perspectives of student-athletes on how much each dimension of wellbeing would influence performance and how important they perceived a state of wellbeing in their performance development. Those items scaled from 1 (*very much)* to 5 (*not at all*).

#### Availability, experience, and expectation of support services

Two items were specified to collect information about the availability of guidelines and support programs on athlete wellbeing and/or mental health within the national sports federation. Furthermore, a total of 25 items were prepared in order to investigate the student-athletes’ experience of receiving support services in relation to their wellbeing. In contrast, one item was added to identify the level of expectation for developing national support services by the government and/or national sports federations. Those items were developed with the perspectives on general service provision in relation to information, detection, proactive and/or reactive support service, tools, and networking.

#### Life satisfaction

The overall satisfaction with life scores from the national wellbeing and quality of life survey were taken on an 11-point scale from 0 (*not satisfied at all*) to 10 (*very satisfied*) to compare the participants’ scores with the general population in Japan (Cabinet Office, 2018).

### Procedures

Ethical approval for this study was granted by the authors’ sports science institute ethical review committee (Reference #047) in accordance with the Declaration of Helsinki. A written informed consent form describing the aim, methods, risks associated with participation, confidentiality considerations, and data ownership and management methods of the study was provided to the student-athletes before the participants filled out the web-based questionnaire. They could withdraw from participation at any time, even after they have agreed to participate in the study. After we obtained informed consent from the participants, they completed the survey using the web-based questionnaire system (Tokyo: Cross Marketing Group Inc.), taking approximately 15–20 min on a confidential and voluntary basis. The survey was conducted from February to March 2021.

### Analysis

The Chi-square tests were used to determine the presence and magnitude of deviations away from expected distributions, and the significance level α was set at 0.05. Correlation analysis was applied to identify the relationship between the items with the following thresholds: < 0.1, *trivial*; 0.1–0.3, *small*; 0.3–0.5, *moderate*; 0.5–0.7, *large*; 0.7–0.9, *very large*; and 0.9–1.0, *almost perfect* (Hopkins et al., 2009). The Statistical Package for Social Science (SPSS) for Windows version 27 (Armonk, NY: IBM Corp.) was used for this analysis. A Welch’s *t*-test was conducted for group comparison using RStudio statistical computing software version 1.4.1717 (Boston, MA: RStudio), and the significance level α was set at 0.05. Uncertainty in true (population) effects values was expressed as 90% confidence limits.

## Results

### The current state of student-athlete wellbeing

The state of the participants’ wellbeing in the past 3 years was perceived as *somewhat good* in all physical (*M* = 1.91, *SD* = 0.94), mental (*M* = 2.05, *SD* = 0.97), educational (*M* = 2.10, *SD* = 1.07), organizational (*M* = 2.42, *SD* = 1.26), social (*M* = 2.06, *SD* = 1.04), financial (*M* = 2.19, *SD* = 1.06), and legal (*M* = 2.53, *SD* = 1.36) dimensions. Among the seven dimensions of wellbeing, the participants self-evaluated their legal wellbeing as the highest, indicating “*relatively not good.” In contrast, physical* wellbeing at the lowest indicated “*relatively good.”* The results of the correlation analysis showed that the overall satisfaction with life scores and the seven dimensions of wellbeing were insignificant (*p* > 0.05). However, the relationship between the overall satisfaction with life and wellbeing scores between the groups showed some significant differences (Table 1). In particular, moderate and small positive correlations were observed between the overall satisfaction with life and wellbeing scores in organizational and financial dimensions only in the elite group (*r* = −0.51, *p* = 0.001; *r* = −0.36, *p* = 0.031, respectively). No significant differences were observed in the sub-elite group.

**TABLE 1:** The relationship between the overall satisfaction with life and wellbeing scores of the participants in the elite athlete group (represented Japan in the senior competition at the international level) and sub-elite athlete group (competed at the national level).

	Overall satisfaction with life	Physical wellbeing	Mental wellbeing	Educational wellbeing	Organizational wellbeing	Social wellbeing	Financial wellbeing	Legal wellbeing
Overall satisfaction with life	–	–0.30	–0.28	0.01	−0.51**	–0.20	−0.36*	–0.22
Physical wellbeing	–0.10	–	0.65**	0.62**	0.69**	0.56**	0.72**	0.30
Mental wellbeing	–0.11	0.89**	–	0.67**	0.68**	0.62**	0.77**	0.55**
Educational wellbeing	–0.18	0.65**	0.67**	–	0.48**	0.63**	0.64**	0.49**
Organizational wellbeing	–0.07	0.43**	0.45**	0.65**	–	0.51**	0.72**	0.51**
Social wellbeing	0.03	0.62**	0.66**	0.77**	0.65**	–	0.55**	0.46**
Financial wellbeing	–0.05	0.66**	0.67**	0.71**	0.61**	0.73**	–	0.66**
Legal wellbeing	0.08	0.45**	0.40**	0.45**	0.65**	0.52**	0.58**	–

Upper level, correlation coefficients of the elite athlete group; lower level, correlation coefficients of the sub-elite athlete group.

*p < 0.05, **p < 0.01.

Furthermore, based on Chi-square tests between states of wellbeing and independent variables, no significant difference was found in gender, place of living, and Olympic sports compared to Paralympic sports. The performance level, however, showed significant differences in organizational (*p* = 0.002), financial (*p* = 0.004), and legal (*p* = 0.004) dimensions of wellbeing. The elite athlete group indicated *not being good at all* in organizational wellbeing (*p* = 0.02) and *not so good* in social wellbeing (*p* = 0.01), whereas *somewhat good* in legal wellbeing (*p* = 0.01) compared to the sub-elite group. Interestingly, only the sub-elite athlete group showed their uncertainty (i.e., *not sure*) about their wellbeing in organizational (*p* = 0.03), social (*p* = 0.004), financial (*p* = 0.04), and legal dimensions (*p* < 0.000). There was no significant difference in the overall satisfaction to life score between the elite and sub-elite athlete groups [*p* = 0.26 (90% confidence limits –1.47 to 0.27)].

Given that this study was conducted in early 2021, the influence of COVID-19 on their wellbeing was observed. As a result, the COVID-19 pandemic was perceived to have an impact on the state of student-athletes’ wellbeing to some degree, as approximately half of the participants indicated either being *greatly influenced* or *somewhat influenced* in physical (57%), mental (61%), educational (52%), organizational (48%), social (48%), financial (49%), and legal (44%) wellbeing.

### Athletes’ perception of the influence and importance of wellbeing for performance

#### Influence on their performance

About half of the participants considered their performance was *greatly influenced* or *somewhat influenced* by physical (56%), mental (53%), educational (50%), social (47%), financial (42%), and legal (38%) wellbeing (Table 2). Moreover, significant differences were observed between the elite and sub-elite groups in social, financial, and legal wellbeing [*p* = 0.03 (90% confidence limits –0.88 to –0.13), *p* = 0.004 (90% confidence limits –1.03 to –0.29), and *p* = 0.003 (90% confidence limits –1.15 to –0.44), respectively]. Thus, it was found that the student-athletes in the elite group perceived their state of wellbeing to affect their performance more influence on performance than the athletes in the sub-elite group.

**TABLE 2:** Athletes’ perception of the influence and importance of wellbeing for performance between the elite athlete group (*n* = 36) and sub-elite athlete group (*n* = 64).

	Influence on performance					Importance for performance				
	Elite group		Sub-elite group			Elite group		Sub-elite group		
Measure	*M*	*SD*	*M*	*SD*	*P*	*M*	*SD*	*M*	*SD*	*P*
Physical wellbeing	2.25	1.05	2.56	1.11		1.89	0.78	1.75	0.89	
Mental wellbeing	2.22	1.02	2.55	1.14		1.92	0.81	1.72	0.81	
Educational wellbeing	2.42	1.08	2.73	1.13		2.11	0.85	2.09	0.83	
Social wellbeing	2.28	1.06	2.78	1.11	*	1.92	0.65	1.88	0.83	
Financial wellbeing	2.25	1.02	2.91	1.15	*	1.97	0.84	1.98	0.86	
Legal wellbeing	2.25	0.97	3.05	1.10	*	2.08	0.81	2.16	0.88	

*p < 0.05.

#### Importance of their performance

Many participants considered the dimensions of physical (83%), mental (80%), educational (72%), social (78%), financial (76%), and legal (71%) wellbeing to be *very important* or s*omewhat important* in relation to improving their own performance (Table 2). No significant difference was observed between the elite and sub-elite athlete groups (*p* > 0.05), meaning that most Japanese student-athletes consider wellbeing important for their performance development regardless of performance level.

### Availability of support policy, guidelines, and programs in national sports federations

The results revealed that Japan’s support systems and programs were rarely available for student-athletes. First, 11.0% of the participants indicated the availability of guidelines on athlete wellbeing and/or mental health from the national sports federations, whereas 35.0% responded “*No*,” and 54.0% showed “*I do not know.*” Second, only 18.0% revealed that their national sports federation has some kind of policy or implementation of it to support the athlete’s wellbeing and/or mental health. In comparison, some national sports federations have policies but no implementation (11.0%). Third, 21% of the participants indicated no policy or actions within the national sports federations, whereas 50.0% did not know the availability.

### Student-athletes’ experience of support for their wellbeing

The results indicated that most of the student-athletes (85.0%) had never received support for their wellbeing. The reasons were identified as (a) a lack of knowledge about how to access those services (49.4%), (b) the lack of information about those services available to them (43.5%), (c) the lack of understanding of the necessity to receive such support (11.8%), and (d) the lack of a service provider from whom they can receive support (10.6%). Interestingly, nine of 15 participants (60.0%) who experienced athlete wellbeing support in the past revealed that they received support from *educational institutions (i.e., high schools and universities)* rather than national sports federations (*n* = 2) or the Japanese Olympic and Paralympic Committees (*n* = 1). The support services the 15 participants received in the past comprised educational programs to gain knowledge and information (46.7%), develop the athletes’ skills such as resilience and/or coping (40.0%), and mental health-related services (40.0%). Individualized consultation (26.7%), as information delivery and education programs, seemed to be necessary.

#### Information

It was found that only 12.0% of the student-athletes knew the word and the meaning of “a*thlete wellbeing*.” In fact, 99.0% of them claimed, in their perception, that the national sports federations had never delivered information about their wellbeing to them. Moreover, 67.0% indicated that they had never obtained and/or gathered information about “*athlete wellbeing.*” For the rest of the participants, the information sources varied from online movie (e.g., YouTube, SNS etc.) (18.0%), national sports federations (12.0%), literature (7.0%), information delivery from entourage (support staffs = 4.0%, coach = 3.0%, teammates = 3.0%, retired athletes = 2.0%), and website of IOC and/or International Sports Federations (IFs) (2.0%).

#### Detection

The results demonstrated the lack of a detection and monitoring system for student-athlete wellbeing. First, 77.0% of the participants responded that they had no experience when national sports federations approached them to understand their state of wellbeing. Despite the relatively low experience of the student-athletes (23.0%), the detailed detection methods utilized by the national sports federations in their approach were also specified as; (a) conversation with the coach and/or experts (11.0%), (b) informal conversation daily (9.0%), (c) utilization of measurement tools (8.0%), (d) individual confirmation from behavior such as continuous absence in training (5.0%), and (e) clinical diagnostic tests (3.0%). Interestingly, however, no participants indicated their experience in utilizing any tool for detection.

#### Help-seeking behavior when faced with a threat or risk

Most participants indicated that they had never witnessed or experienced behavior that could be considered a threat or risk to the student-athlete’s wellbeing and/or mental health (84.0%). Among 16 participants who have witnessed or experienced inappropriate behavior, 31.2% of those shared or reported it to someone else, such as teammates or team staff (*n* = 6), and the national hotline set by the national sports federations, Japanese Olympic Committee, Japanese Paralympic Committee, or JSC (*n* = 4). The reason why the majority of the student-athletes (68.8%) did not share or report the case was that the athletes; (a) did not want to make it a big deal (45.5%), (b) were afraid of who reported (36.4%), (c) did not know whom to report to (18.2%), or (d) did not want to be involved in (18.2%). Of those who shared or reported it to someone else, however, 60.0% indicated their positive experience by expressing their satisfaction with the correspondence to the issues. Finally, the results demonstrated the lack of information and knowledge about the availability of a hotline, as 75.0% of the participants responded that they had never heard or been aware of the availability of a hotline.

#### Help-seeking behavior when anxious or distressed

The results also showed that 55.0% of the participants had someone whom they could talk to whenever they were anxious or distressed, including parents (61.8%), friends (60.0%), teammates (30.9%), significant others (25.5%), senior athletes (23.6%), brothers and sisters (18.2%), coaches (10.9%), and/or support service staff (5.5%). However, only 19.0% choose to approach experts to seek help. Those experts included psychiatrists (26.3%), clinical psychologists (21.1%), other psychological specialists (e.g., industry and school counselors) (15.8%), sports counselors (15.8%), and so on. Interestingly, 31.6% of those who sought help from experts identified with a coach. Their experience of working with the experts tended to be somewhat positive, as 47.4% indicated their satisfaction, whereas the same number of participants were not sure whether they were satisfied or not. Interestingly, the barriers to not seeking help from experts were identified; (a) lack of knowledge about where they could find the appropriate experts (37.0%), (b) uncertainty about the cost of receiving support (35.6%), (c) disbelief in the ability of experts to solve their problems (30.1%), (d) no clarity about whom to talk to (23.3%), (f) worries about eyes around them (17.8%), and/or (g) a feeling of embarrassment to seek help (12.3%).

### Athletes’ expectations for the national support system and service programs for their wellbeing

**FIGURE 1:**
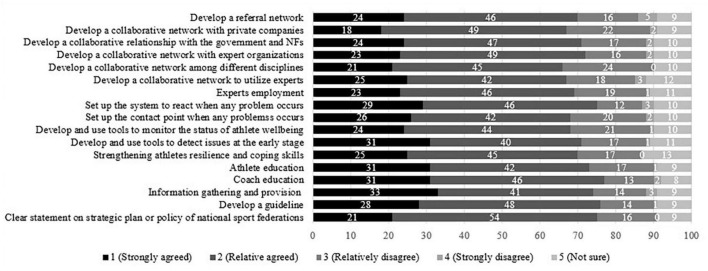
The participants’ expectations for the national support system and service programs on wellbeing on a 5-point scale (1 = *strongly agreed*, 2 = *relatively agreed*, 3 = *relatively disagree*, 4 = *strongly disagree*, 5 = *not sure*).

If the government and national sports federations were to develop the support system and service programs in Japan, 38.0% of the participants expressed their willingness to receive support, while 31.0% were reluctant to use the service in the future. The majority of the participants, however, agreed with the importance and necessity of the government and national sports federations developing the system and programs to promote and support athlete wellbeing in Japan (Figure 1). Based on the results, “*coach education*” was the most expected action (77.0%), followed by “*develop a guideline*” (76.0%), “*clear statement on strategic plan or policy of national sports federations*” (75.0%), and “*set up the system to react when any problem occurs (investigation, measures, and penalties, etc.)*” (75.0%). These results might indicate the need for coaches to understand the field of wellbeing while expecting the government and national sports federations to provide guidance. Considering that all items were somewhat equally supported and even the least expected item obtained 66.0%, it could be concluded that various actions could potentially be taken to develop the national support systems and programs in the future.

## Discussion and practical implications

As there is convincing evidence that indicates pursuing excellence i nhigh-performance sports is associated with various factors that may become threats to the holistic wellbeing of athletes (MacAuley, 2012; Gouttebarge et al., 2019; Giles et al., 2020; Bennie et al., 2021), several high-profile countries in the Olympic and Paralympic Games, such as Canada, Australia, Netherlands, New Zealand, the United Kingdom, the United States, and so on, have started developing their own support systems and programs for athletes to pursue excellence both in performance and wellbeing in recent years. Japan is considered one of the world’s leading countries in high-performance sports by placing in the top 3 in the summer Olympic Games of Tokyo 2020. However, little literature is available in the Japanese context (Kinugasa et al., 2021). As an initial investigation, this pilot study aimed to reveal the general trends of athlete wellbeing in Japan, particularly from the perspectives of university student-athletes. In the following, the discussion was carried out as per the four specific objectives of this study.

First, this study aimed to investigate the current state of student-athlete wellbeing from a holistic development perspective (Wylleman, 2019). Based on the results, the Japanese university students demonstrated a relatively good state in all seven dimensions of wellbeing (i.e., physical, mental, organizational, social, educational, financial, and legal) despite the observation of COVID-19 influence to a certain degree. In fact, the overall satisfaction with life scores of the participants and the general population in Japan were similar (5.7 and 5.9, respectively) (Cabinet Office, 2018). The lower score of wellbeing in organizational and social wellbeing for the elite group somewhat supported the idea that elite athletes need more support than non-elite athletes as they face higher demands that may threaten their wellbeing. In addition, the results show that only the sub-elite athlete group indicates their uncertainty (i.e., *not sure*) about their wellbeing in organizational, social, financial, and legal dimensions, suggesting lower awareness of their wellbeing at the non-elite level. Those results implied that elite athletes need more support for their wellbeing. The holistic approach is preferable by providing not only physical and mental but also social and organizational dimensions of their wellbeing.

The second objective of this study was to understand the level of knowledge about athlete wellbeing in university student-athletes. Given the little information available in Japanese, the result showed that the student-athletes were previously not familiar with the word “athlete wellbeing,” and the majority did not exactly know the meaning of it. However, given the description in the written form attached to the survey, approximately half of the student-athletes perceived their performance was significantly influenced or somewhat influenced by physical, mental, educational, social, and financial wellbeing. Moreover, more than 70.0% of the participants considered athlete wellbeing in all dimensions to be very important or somewhat important to improving their performance. These results have implications in two ways. One is that it is essential to raise awareness of athlete wellbeing in Japan so that athletes recognize the importance of self-care for their wellbeing, which, in turn, influences their performance. The other one is that those involved in the field of wellbeing should not take wellbeing apart from performance by understanding that those two are intercorrelated, at least from the perspective of student-athletes. In other words, the support for athlete wellbeing should be designed to align with the performance development plan and progress of the athletes.

The third objective of this study was to reveal the athlete’s perception of the availability of a support system within the national sports federation. In regards to the availability of policies, guidelines, and programs, the results suggested that (a) there were only a few national sports federations already accommodating the support policies, programs, and guidelines in their systems, and (b) the information might not be appropriately delivered to athletes despite the availability, or (c) the athletes were not eligible to access the service and information due to their performance level. As only 2% of the participants indicated their experience of receiving support services for their wellbeing from the national sports federation, it could be argued that few national sports federations obtain the support system within the organization supporting point (a) indicated above. Given that the results derived from the athletes’ perception, however, further investigation of the national sports federation is necessary to conclude that they have not developed the policy, guidelines, and service programs for their athletes.

The fourth objective was to investigate athletes’ experiences of support services from various points of view, including information, detection, and seeking behavior in reacting to a threat and/or risk, as well as a feeling of anxiety and/or distress. Overall, the results proved that most of the university student-athletes had never experienced, at least in their recognition, receiving support services for their wellbeing in the past. In terms of information, 67.0% indicated that they had never obtained and/or gathered information about “athlete wellbeing.” Interestingly, however, it was found that the lack of information about the support service available and where to access it was the number one reason cited by the student-athletes, rather than their rejection of the service. Despite the small sample size who obtained the information about wellbeing (33.0%), given the results indicating their behavior to seek information about wellbeing, it could be recommended to consider the use of an online platform such as YouTube and/or social networking sites (18.0%) in addition to the national sports federations (12.0%) and entourage (e.g., coaches, teammates, support staff, former athletes) (12.0%) as the channel for information delivery. Nevertheless, it should be cautious about the accuracy of the information, as only 2.0% indicated their experience of seeking information on the official website of IOC and/or Ifs. In order for student-athletes to systematically access the right information in the Japanese language, a somewhat “one-stop-shop resource center” could be a possible action, while conducting further research in the Japanese context is necessary to provide evidence for policy-makers and practitioners. Kinugasa et al. (2021) stated the definition of athlete wellbeing in Japanese, which could be used in policy and practice in the future. Regarding detecting problems associated with athlete wellbeing, the results showed that 77.0% of them had no experience of receiving this service from national sports federations. As for detection techniques, it was found that communication and/or interaction was more commonly used than the application of measurement tools and/or clinical tests. Furthermore, concerning help-seeking behavior, 84.0% claimed no experience of facing or witnessing inappropriate behavior that could be a threat or risk to the athlete’s wellbeing. Among those 16.0% with experience, approximately 70.0% did not share or report it to someone because they did not want to make it a big deal (45.5%) and/or were afraid of who reported it (36.4%). Despite the availability of a hotline for wellbeing in a broader sense, only 4 participants have used it to report the problem. This was probably due to the lack of awareness, as 75.0% of the participants indicated that they had never heard of or been aware of the hotline. It was evident that the student-athletes tended to first report problems to their entourage rather than the official hotline set by organizations to seek help. Finally, the results indicated that 45.0% of the student-athletes did not have anyone to talk to about their anxiety or distress. Within the 55.0% of the student-athletes, it was found that approximately 60.0% of them would initially talk to their parents or friends rather than coaches or support staff. It implied that information and education to athletes and coaches are not enough but include parents and entourage to understand athlete wellbeing better. Additionally, despite the low rate of student-athletes (19.0%), coaches (31.6%), psychiatrists (26.3%), and clinical psychologists (21.1%) were the top three experts from whom student-athletes have sought help in the past, while non-psychology experts such as medical doctors and athletic trainers/physiotherapists (10.5%) were also indicated for their options. These results indicate that it is essential for the organization to consider the development of a network with experts in the fields of mainstream psychology and medicine, as well as the involvement of coaches within the support system in Japan. It should, however, be noted that only 8.0% of the student-athletes indicated their willingness to talk to experts, while 43.0% did not feel a need, and 30.0% could not seek help despite wanting to do so. Interestingly, the main barriers for the student-athletes were a lack of knowledge about where they could find the appropriate experts, their uncertainty and a feeling of incapability about the cost, and their distrust in the ability of experts to solve their problems. According to these results, it could be suggested that to facilitate the change in athletes’ help-seeking behavior from experts, information and education, as well as a reference network to access the appropriate experts for their issues, are necessary as the barriers seemed not to be the stigma often associated with athletes.

These findings could then lead to a discussion about the implications associated with this study’s last objective, which was to identify the types of support student-athletes expect from the government and/or national sports federations in the future. It was interesting that most student-athletes strongly or relatively agreed to all of the proposed actions, including clear guidance of the direction, information gathering and delivery, athlete and coach education, the development of detection and monitoring tools, the settling of the system to react to problem occurrences, the employment of experts, and the development of a collaborative network system with experts, expert organizations, private companies, government, and national sports federations, and the development of a referral network. These results somewhat supported the argument that to implement policy into practice, increasing awareness and knowledge through information delivery is essential but not sufficient to address athletes’ various needs for mental health and wellbeing (Purcell et al., 2019). The development of these support frameworks could be considered the common approach in a national system worldwide (Department for Digital, Culture, Media and Sport, 2018; Moesch et al., 2018; Australian Institute of Sport, 2020; High Performance Sport New Zealand, 2021). As those approaches were somewhat equally agreed upon (Figure 1), however, it was difficult to make the prioritization among those actions in this pilot work. Interestingly, however, more than one in three athletes showed resistance to receiving support services even if the government and/or national sports federations establish those support frameworks in the high-performance sport system. These attitudes might be associated with a lack of knowledge and information, as observed in their experience of receiving support from experts rather than cultural stigma. Therefore, promoting athlete wellbeing is necessary to consider those obstacles when designing and planning the development of policies, systems, and programs to support athlete mental health and/or wellbeing to facilitate its utilization in better ways.

In summary, this pilot study of university student-athlete wellbeing in Japan revealed the general trends in broader and holistic perspectives as little information was available. Based on the results, the current state of student-athletes’ wellbeing was relatively positive despite the influence of COVID-19. Given the lack of information related to athlete wellbeing in Japan, the student-athletes demonstrated low recognition of the word and meaning of “*athlete wellbeing*.” They indicated, however, that they perceived their state of wellbeing might influence their performance and, therefore, be important for their performance development. Nevertheless, in the perception of student-athletes, few national sports federations have policies, guidelines, and support programs in place for athletes. It was, therefore, evident that most of the student-athletes had never experienced the support service on wellbeing in terms of information, detection, and help-seeking behavior. Despite the uncertainty of utilizing the support provided, the student-athletes agreed that it was necessary for the government and/or national sports federations to take actions such as clear guidance of the direction, information gathering and delivery, athlete and coach education, the development of detection and monitoring tools, the settlement of the system to react to problem occurrences, employment of experts, and the development of a collaborative network system with experts, expert organizations, private companies, government and national sports federations, and the development of a referral network. Given these results, further investigations were required, particularly targeting athletes in high-performance sports (i.e., Olympic and Paralympic athletes) and national sports federations.

## Limitations and future direction

There were some limitations associated with this pilot study. First, the COVID-19 pandemic affected the findings as the study was conducted during the State of Emergency in Japan. In fact, approximately half of the participants perceived the influence of the COVID-19 pandemic on their wellbeing. To account for the COVID-19 pandemic, the states of athlete wellbeing in each dimension were asked over the past 3 years. Since this investigation focused on the general trend of student-athletes’ perceptions of their state and environment of holistic wellbeing, the instrument consisted of only one set of items specifically capturing the influence of COVID-19. Second, in terms of methodology, the sample size of 100 is limited for subgroup analysis. Therefore, further studies could suggest that an in-depth analysis of athlete wellbeing, such as gender, length of time in the field, and status of physical limitations, with larger sample size, might support assuming the generalizability of the study. Finally, as the interval of 7 days for test-retest reliability might not be sufficient, a minimum time gap of a fortnight may be necessary for future investigation.

Based on the findings from this pilot study, further investigation should be carried out to develop the national support system in Japan. First, future studies could target elite athletes (i.e., Olympic and Paralympic athletes) on a larger scale. Second, as the findings were only derived from athletes’ perspectives, it could suggest investigating the national sports federation’s point of view regarding the availability of athlete support systems and/or programs. Third, the researchers could consider the study about the wellbeing of the entourage because the issues and challenges associated with the topic of wellbeing are not necessarily limited to athletes as they also spend considerable time in a highly demanding environment (Breslin et al., 2017).

Given the lack of information on the Asian population in the field of athlete well-being and mental health was evident (Reardon et al., 2019), international collaborative research in the Asian region is necessary. Furthermore, comparing Asian and Western countries could help in cultural considerations in developing each country’s policies, systems, and programs. As the JSC, the mother organization of the JISS, is the only national sports agency responsible for grassroots to high-performance sports in Japan, the social science research group of the JISS will continue to study in this field to provide further evidence and information to support policy implementation in the field of athlete wellbeing by collaborating with researchers both in Asia and the world in the future.

## Data availability statement

The datasets generated for this study will not be available due to the privacy of the participants. Please contact the corresponding author for any further information and any requests to access the datasets.

## Ethics statement

The studies involving human participants were reviewed and approved by Japan Institute of Sports Sciences Ethical review Committee. The patients/participants provided their written informed consent to participate in this study.

## Author contributions

YN conceptualized the study, developed the instrument, supervised the analysis, and drafted the manuscript from initial to final. CK conducted data analysis and drafted parts of the methods, measures, and results. TK supervised the whole process as the project leader, recruited participants, conducted the survey and data analysis, and drafted some of the methods, measures, and results in parts. All authors contributed to the article and approved the submitted version.
